# The Agricultural Green Production following the Technological Progress: Evidence from China

**DOI:** 10.3390/ijerph19169876

**Published:** 2022-08-10

**Authors:** Shuxing Xiao, Zuxin He, Weikun Zhang, Xiaoming Qin

**Affiliations:** 1 School of Public Administration and Law, Hunan Agricultural University, Changsha 410128, China; 2 School of Teacher Education, Shaoguan University, Shaoguan 512005, China; 3 School of Economics, Guangdong University of Finance & Economics, Guangzhou 510320, China; 4 School of Social and Public Administration, Lingnan Normal University, Zhanjiang 524088, China; 5 Guangdong Provincial Key Laboratory of Aquatic Products Processing and Safety, Guangdong Ocean University, Zhanjiang 524088, China

**Keywords:** agricultural green production, technological progress, green total factor productivity, spillover effect, threshold effect

## Abstract

This study performs the spatial Durbin model (SDM) and threshold model to analyze the efficiency of agricultural green production following technological progress from 1998 through 2019. The SDM supports a nonlinear contribution of technological progress spillover to agricultural green total factor productivity (GTFP), exacerbated by upgrading agricultural structure. Moreover, the threshold model confirms that technological progress has a single threshold effect on agricultural GTFP with the rationalization of the agrarian system as a threshold variable; meanwhile, the contribution of technological progress to agricultural GTFP is less than that of agricultural total factor productivity. Out of the expanded application of dissipative structure theory in agricultural GTFP systems innovatively, this study reveals the urgency to strengthen the innovation of independent technology, lower the threshold for introducing technology, and optimize the agrarian structure in the long-term sustainable agriculture for the economies that are undergoing a similar development stage as China.

## 1. Introduction

The development of green agriculture is the only way for all countries and regions to achieve sustainable agricultural development. According to the United Nations Department of Economic and Social Affairs, the population over the world will exceed the 10 billion mark by 2050, and it is believed that the supporting grain growth must reach 70%. Inevitably, the consumption of natural resources has increased under the enormous pressure of agricultural production, accompanied by environmental problems, such as soil contamination, haze, and fresh-water pollution. The increasingly rigorous conditions, such as temperature extremes, water scarcity, and the reduction in arable lands represent a lurking threat to human society [1]. So how do we deal with these potential problems? China has elevated ecological progress to an unprecedented strategic position and made plans to promote green agricultural development. It believes agricultural development should be transformed from quantitative expansion to quality improvement [2]. In the case that other objective conditions cannot be changed, technological progress can promote the transformation of agricultural production mode and improve the green production efficiency of agriculture, which is conducive to the formulation of targeted, sustainable agricultural technology policies.

The “green development” proposed by the United Nations Development Program in 2002 has been widely accepted as the only way to realize the organic unity of environmental protection and economic development. In this case, Oskam first incorporated the unexpected output into the agricultural total factor productivity measurement, and then Li and Xu proposed the green total factor productivity [3]. As a further extension of the concept of sustainable development, agricultural green total factor productivity (GTFP) incorporates resource and environmental elements into the measurement system to check the effectiveness of green agriculture [4]. Unlike traditional total factor productivity (TFP), the agricultural GTFP system belongs to the category of nonspatial structure. Although offsetting wrong output leads to energy dissipation, open agricultural GTFP systems exchange matter and energy with their external environments, which may create a new state of order once the external conditions reach a certain threshold. So far, in practice, technological progress has made it possible to promote agricultural GTFP by reducing required inputs, improving productivity, and achieving sustainability, profitability, and productivity in the agricultural sector [5,6]. In particular, a continuous exchange of materials or energy with the outside through technological progress reduces carbon emissions [2,7], thus improving agricultural GTFP.

Technological progress may be neglected in promoting the improvement of agricultural GTFP. Few studies have focused on the importance of technological progress on agricultural GTFP, especially the impact of agricultural technology spillover on agricultural GTFP. And, once the marginal benefit exceeds the marginal import cost, technology spillover will promote the growth of agricultural GTFP. On the contrary, technology spillovers will hamper its growth. In other words, the effect of agricultural technology spillover on agricultural GTFP is nonlinear.

Most importantly, during the 13th Five-Year Plan period, by spreading critical technologies in 13 major grain-producing areas across the country, grain output increased by 8.5 percent, utilization rates of water and fertilizer increased by 14.7 percent, utilization rates of light and heat increased by 16.6 percent, labor productivity increased by 31 percent, fertilizer application decreased by 20 percent, and pesticide application decreased by 30 percent. At the same time, China has set up 109 agricultural nonpoint source pollution control demonstration zones and 110 waste treatment demonstration projects, which have reduced the pollution load of demonstration zones by 30 percent and the availability of heavy metals in cultivated land by 50 percent [8,9,10]. As a further extension of the concept of sustainable development, agricultural GTFP incorporates resources and environmental factors into the measurement system to check the effectiveness of green agriculture [4]. Therefore, technological progress should be considered when studying the resources and environmental aspects of agricultural GTFP.

This study takes agricultural GTFP as the research object. The Spatial Durbin and Threshold Models were used to study the efficiency of green agricultural production after technological progress from 1998 to 2019. According to the research results, the spillover of technological progress is a nonlinear contribution to agricultural GTFP, and the donation is intensified by technological progress to agricultural structure upgrading. The threshold model confirmed that technological progress had a single threshold effect on agricultural GTFP, and rationalization of the farm structure was the threshold variable. In addition, the dissipative theory of the agricultural GTFP system is extended theoretically, and the urgency of strengthening independent technological innovation to promote green agricultural production is revealed.

The main contributions of this paper are as follows: Firstly, this paper analyzes the changes in agricultural green production after technological progress from the perspective of green total factor productivity. Secondly, this paper examines the spatial spillover effect of technological progress and explores the impact of technological progress on green agricultural production in neighboring areas. Finally, this paper considers the threshold effect of technological progress.

## 2. Literature Review and Theoretical Framework

The value of agricultural technology must be realized through spatial diffusion. The earliest introduction of spatial factors to discuss the phenomenon of technology diffusion is Haugerstrand, who believes that spatial distance is the primary resistance to information flow in the process of technology diffusion [11]. Later, the spatial spillover of technological progress has been regarded as a significant engine for sustainability. In recent years, even obtaining a great deal of theoretical or empirical results on whether the technology spillovers can effectively promote sustainability in inflow regions has not reached a unified conclusion. The impact of technology spillovers on sustainability is mainly reflected in productivity, pollutant emission reduction, etc. Jiao et al. showed that every green technology innovation level and technology spillover capacity was increased by 1%, and the carbon intensity was reduced by 0.1303% and 0.1558%, respectively [12]. Zhai et al. found that technology has a significant positive impact on the green conversion efficiency of local provinces. In contrast, the spillover effect of technology R&D on the green conversion efficiency of neighboring provinces is significantly negative, and the spillover effect of technology commercialization is extremely positive [13]. Huang et al. suggested that the technology spillovers through openness benefit China’s total factor productivity [14]. Baniasadi et al. believed that the existence of international technology spillover has had a favorable impact on the TFP growth of the agricultural sector of Iran [15].

The other scholars believed that technology spillover is not significantly positive in promoting sustainability as we expected. Liu et al. found that the technology spillover does not bring a pollution halo effect to local areas but to adjacent regions [16]. Pan et al. believed that the decreasing impact of green technology spillover on energy intensity depends on the absorptive capacity of each province and the period, and there is a threshold effect of green technology spillover on energy intensity in China [17]. Zhao et al. believe that independent innovation has no inhibiting effect on haze pollution, and technology introduction has aggravated haze pollution to a certain extent [18]. Ratinger et al. confirmed that whereas the domestic agricultural technologies played a positive role in agricultural productivity, the results for the agrarian technologies spillovers are relatively weak [19]. Such seemingly contradictory effects have given rise to skepticism about whether agricultural technology spillovers should be improved to increase sustainable agricultural growth. The studies mentioned above on the relationship between the spillover of technologies and sustainability mainly involve carbon emissions from various sectors, TFP of agricultural sector and industry, but there is less research on GTFP of the farming sector. Unlike TFP, considering the input and output of undesirable factors in the agricultural production processes, agricultural GTFP is an important engine for sustainable agricultural growth. Green development and ecological civilization construction are essential issues in China’s ecological and environmental protection undertakings [20].

We focus on the spillover effect of technological progress on agricultural GTFP in China with the consideration of the agrarian structure. Is it in a linear regime? How about the contribution of technological advancement to agricultural GTFP in China, taking the farm structure as a threshold variable? And we are also wondering about the threshold effect on the farming TFP.

The connotation of the agricultural GTFP system indicates that it is virtually a dissipative structure. Incorporating the undesired output into agricultural TFP measurement, the innovative design will be rebuilt to reduce undesired input and work during the interaction between the agricultural GTFP with its exterior or among the internal elements. Since constantly exchanging material and energy with the external environment and continuously drawing “negative entropy” from the internal and external environment to offset its own “entropy,” the energy dissipation of the agricultural GTFP system will inevitably lead to being smaller than the agricultural TFP. According to Deng et al., the rural system in China belongs to the dissipative structure category [21]; the agricultural GTFP system is one subsystem of the agrarian system; thereby, it is rational to clarify the effect of agricultural structure on the contribution of technological progress to agricultural GTFP in China base on an analytical framework of the dissipation theory. We summarize the characteristics of the agricultural GTFP system as follows:

(a) Open System. The agricultural GTFP system not only absorbs the energy from its internal but also exchange the information with its exterior [21]. The source of agricultural technology could be divided into independent technologies and technologies introduction [22,23]. Naturally biased technical causes lack reproducibility [24,25]. Indeed, the drive for innovation itself can lead to bias by demanding novel methods that will, by definition, not be yet applied elsewhere [26]. Moreover, capital-enhanced and labor-enhanced technological progress further induces changes in the relative endowments and accumulation status of agricultural production factors, thus playing a fundamental decisive role in structural changes [27]. Only when the direction of technological progress matches the factor endowment of the economic unit will it effectively promote the improvement of its efficiency and productivity; Otherwise, it will replicate those procedures blindly and inhibit the growth of reproducibility at the end [28,29,30]. Generally, the endogenous technology in the agricultural system can accurately improve the quality of agriculture. However, due to issues such as space friction and lagging effectiveness, the transplantation, and application of external technology cannot enhance agricultural GTFP effectively.
(b) Nonlinearity Regime. The competitive or cooperative relationships among factors, between factors and the system, and between systems with their exterior contribute to a nonlinearity regime in agricultural GTFP over time and space [21]. The practical application of technology to agriculture is a dynamic process of self-improvement. Once the technologies reach a specific threshold value, they show a variable rate [31], and the agricultural GTFP could mutate to a nonlinearity regime. When the marginal benefit is greater than the marginal cost, technological progress will promote the growth of local agricultural GTFP, but such promotion will attenuate with cost redundancy. On the contrary, the cost of technology introduction will make the marginal cost more significant than the marginal benefit at first. Technology introduction hinders the growth of agricultural GTFP, but that hindrance decays as the cost is amortized. This is also in line with the law of diminishing marginal returns.
(c) Far from Equilibrium. The diverse states of the agricultural GTFP system are the final result of the interaction between the farming GTFP system and its exterior, along with its internal elements [21]. At the temporal scale, the sharing of technology R&D costs, the accumulation of utility, and the improvement of the coordination within the agricultural structure, as well as the agglomeration and diffusion of industries, will all drive the dynamic progress of the agricultural GTFP system. On the spatial scale, technology spillovers and the optimization of the supportive environment for agriculture are the critical factors for the growth of agricultural GTFP [32,33]. The agricultural GTFP system is subject to technologies and agrarian structure. The existence of a sustainable economy depends on technological progress. In addition, technological progress is closely related to upgrading industrial networks [34,35].

The upgrading of agricultural structure, as the name implies, is to excavate, integrate and utilize the practical resources of agriculture (such as capital, workforce, natural resources, science, technology, etc.), optimize the allocation of various resources, improve the degree of marketization of agriculture, and increase farmers’ income. The rationalization of the agricultural structure mainly refers to the reasonable proportion of agriculture, forestry, animal husbandry, and fishery, making full use of practical resources, and the rural industries complement each other and develop together. In contrast, the advanced agricultural structure emphasizes the improvement of the overall level. In terms of essential requirements, the upgrading of the agrarian system is mainly for the coordinated development of industries and mutual promotion, not only to solve the constraints of the development of relatively backward sectors but also to prevent the duplication of construction of specific departments and cause the waste of resources, that is, the various departments of agriculture must strengthen the communication, optimize the allocation of multiple resources, and coordination. Avoid waste of resources and abnormal development of agriculture. This shows that upgrading the agrarian structure is in line with the requirements of technological progress and the growth of the farming GTFP. In essence, upgrading the agrarian system mainly refers to transforming the agrarian structure from low-level to high-level by using various resources such as human resources, financial resources, and national policies. The essence of technological progress and the growth of the agricultural GTFP is to maintain the balance between economic growth and the ecological environment so that the social economy, population, nature, resources, etc., can develop in a coordinated and sustainable manner. Following this logic, upgrading the agricultural structure could accelerate the impact of technological progress on agricultural GTFP.

Existing literature laid important references or inspirations for the study. However, the scholars mentioned above lack in-depth consideration of the dissipative structure of the agricultural GTFP system, and they did not discuss the overall characteristics of the effect of agrarian structure on the contribution of technological progress to agricultural GTFP in China. Agricultural activities have more substantial geographical restrictions than industry, and agricultural technology has a more significant time lag in return and greater friction in diffusion space. The agricultural GTFP system exhibits a series of characteristics, i.e., an opening system, being far from equilibrium, and a nonlinearity regime. An analysis framework based on the dissipation theory may more accurately evaluate the impact of agricultural structure on the contribution of technological progress to agricultural GTFP and has more policy implications to study further.

Given this, the remainder of the paper is structured as follows. Firstly, we measure the technological progress by the Solow growth model and calculate the agricultural GTFP by a nonradial and nonoriented stochastic block model (SBM), respectively. We also divide the upgrading of agrarian structure into two aspects: rationalization and advanced agricultural structure. Next, we perform a spatial Durbin model to identify the nonlinear impact of technological progress spillover on agricultural GTFP with the agrarian structure as an influencing factor, and then construct a threshold panel model to explore the threshold effect of technological progress on agricultural GTFP when an agricultural structure is used as a threshold variable, and further discuss the difference between agricultural GTFP and TFP, to highlight the energy dissipation of agricultural GTFP. Finally, we discuss results concerning the existing literature and draw policy conclusions.

## 3. Methodology

### 3.1. Methods

#### 3.1.1. The Calculation Method of the Agricultural Technological Progress

Comparatively, the regression method based on a sufficient theoretical basis is more objective and can effectively avoid artificial estimation than the empirical method. Referring to Zheng et al. [36] and Xu et al. [37], the Solow growth model can be extended based on the conventional Solow framework to infer the agricultural technological progress as follows:

(1) $$Y_{t}=A_{t}F(k_{1},k_{2},k_{3},k_{4})=A_{t}k_{1}^{\beta_{1}^{}}k_{2}^{\beta_{2}^{}}k_{3}^{\beta_{3}^{}}k_{4}^{\beta_{4}^{}}$$

where $Y_{t}$ is the value of agricultural production for each province; $A_{t}$ is the indicator of the technological progress, $k_{1}$, $k_{2}$, $k_{3}$ and $k_{4}$ are the arable land for agriculture, rural labor force, total power of agricultural machinery, and fertilizer for agriculture tons of quantity, respectively; and $\beta_{1}$,$\beta_{2}$,$\beta_{3}$, and $\beta_{4}$ are the proportions of former variables. Suppose the returns to scale is constant, that is, $\beta_{1}+\beta_{2}+\beta_{3}+\beta_{4}=1$. Further, the formula for calculating technological progress is:

(2) $$A_{t}=\frac{Y_{t}}{k_{1}^{\beta_{1}}k_{2}^{\beta_{2}}k_{3}^{\beta_{3}}k_{4}^{\beta_{4}}}$$

Next, we will further determine the values $\beta_{i}$. Multiple linear regression analyses using Stata 15.1 software by the StataCorp (College Station, TX, USA), can be performed to obtain the estimated values $\beta_{i}$. Consequently, we obtain $\beta_{1}=0.204$, $\beta_{2}=0.036$, $\beta_{3}=0.189$, and $\beta_{4}=0.570$. Finally, we put these parameters into Formula (2) to obtain the technological progress smoothly.

#### 3.1.2. The Calculation Method of the Upgrading of Agricultural Structure

The upgrading of agricultural structure mainly refers to the development process in which agricultural technology level, management experience, and production conditions gradually realize Pareto improvement and promote the continuous improvement of various production factors and human capital. The rationalization of the agricultural structure mainly refers to the reasonable proportion of agriculture, forestry, animal husbandry, and fishery, making full use of practical resources, and the rural industries complement each other and develop together. In contrast, the advanced agricultural structure emphasizes the improvement of the overall level. Scholars generally measure the rationalization of a farming structure by the Theil index (TL). The measure formula is as follows:

(3) $$\mathrm{TL}=\sum_{i=1}^{n}\left(\frac{y_{i}}{y}\right)\ln\left(\frac{y_{i}/y}{l_{i}/l}\right)$$

In Equation (3), y represents the output value of agriculture, forestry, animal husbandry, and fishery; $y_{i}$ is the total output value of various industries (agriculture, forestry, animal husbandry, and fishing); l represents the number of employees in each industry; and $l_{i}$ is the number of the labor force in the agricultural sector. The larger TL is, the worse the coordination degree among agricultural subsectors is; that is, the more unreasonable the farm production structure is. On the contrary, it tends to be reasonable. In particular, when the labor productivity of each agricultural subdivision sector is consistent with the average agricultural labor productivity, TL = 0, reaching an equilibrium state [38]. It should be noted that since China’s official statistics do not release data on the number of the labor force in each industrial segment of the agricultural sector, the practice commonly used in academia is to take the proportion of the output value of each industrial part in the total output value in each year to make up for this defect.

Concerning the availability of data and the comprehensive nature of the indicators, this paper adopts the entropy method to measure the advancement of an agricultural structure by the variables, including the ratio of agriculture and animal husbandry in output value, the area ratio of grain to crop, the output value of forestry, animal, husbandry and fishery industry, and the ratio of each agricultural sector in total output value.

#### 3.1.3. The Calculation Method of the Agricultural GTFP

To overcome the inaccurate measurement results and rank multiple decision units [39], this paper introduces the nonradial and nonoriented SBM to calculate the farm GTFP and the input and output variables listed in Table 1.

Unlike TFP, agricultural GTFP considers undesired input and output, such as resource consumption and environmental costs. This article insists that agricultural carbon emissions mainly come from six parts, i.e., chemical fertilizer, pesticides, agricultural film, machinery, agricultural plowing, and irrigation [40]. The estimation formula of agrarian carbon emissions is as follows:

(4) $$E=\sum E_{i}^{}=\sum T_{i}^{}*\delta_{i}^{}$$

In Formula (4), E represents the total amount of agricultural total carbon emission, $E_{i}^{}$ is the carbon emissions of various carbon sources, $T_{i}^{}$ represents the amount of carbon emission source, and represents $\delta_{i}^{}$ the carbon emission coefficient of each carbon emission source. Li et al. [40] summarized the agricultural carbon emission as follows: the carbon emission coefficients of chemical fertilizer, diesel oil, pesticide, agricultural film, tillage, and agricultural irrigation were 0.895 6 kg·${\mathrm{kg}}_{}^{-1}$, 0.5927 kg·${\mathrm{kg}}_{}^{-1}$, 4.934 1 kg·${\mathrm{kg}}_{}^{-1}$, 5.18 kg·${\mathrm{kg}}_{}^{-1}$, 312.6 kg·${\mathrm{km}}_{}^{-2}$, and 20.476 kg/${\mathrm{hm}}_{}^{2}$, respectively. It is worth noting that this paper defines agricultural productivity without considering carbon emissions as agricultural TFP (ATFP).

### 3.2. Sample and Data Collection

Aiming to analyze the efficiency of agricultural GTFP in China, this study spans 1998 to 2019 and focuses on 31 administrative provinces in mainland China, excluding Hong Kong, Macao, and Taiwan. The panel data set with the yearly frequency issued from China Statistical Yearbook from 1997 to 2020, China Rural Statistical Yearbook from 1997 to 2020, China Population Yearbook from 1997 to 2020, and Employment Statistical Yearbook from 1997 to 2020, and the China Stock Market and Accounting Research Database. The missing values of relevant indicators in the yearbook are filled in by interpolation.

### 3.3. Spillover Estimation

#### 3.3.1. Preliminary Analysis of the Spatial Durbin Model

We detect a spatial autocorrelation by utilizing the geographical adjacency spatial weight matrix $w_{\mathrm{ij}}$. The analysis in Table 2 shows that there is a spatial correlation between the upgrading of agricultural structure, including the rationality (TL) and advancement (EI), technological progress (AST), and agricultural GTFP (AGTFP) in China. The technological progress and agricultural structure manifest significant spatial agglomeration, while the AGTFP shows a fluctuating spatial correlation. Noteworthy, Moran’s index of AGTFP is significantly positive in all the years, except for the two-time spans of 2001–2004 and 2018–2019. According to Table 2, all four Core variables have significant spatial autocorrelation. Therefore, the analysis with models that capture these spatial features should be considered.

#### 3.3.2. The Specification of the Spatial Durbin Model

To study the spatiotemporal characteristics of the nonlinear impact of technological progress on AGTFP, we construct the following spatial regression models by incorporating the spatial lags. Herein, the subscript i represents each province, and subscript t represents the year. $AGTFP_{it}$ defines the AGTFP in i province t year, the explanatory variables in the model are the technological progress (AST), the quadratic term of agricultural technological progress (AST^2^), rationalization of agrarian structure (TL), advancement of am agrarian system (EI), and the interaction terms of the two-by-two multiplication of the above variables. AST and wAST denote each province’s technological advancement and neighboring regions, and the specific meaning of variables of similar form can be derived by analogy. $control_{it}$ and $wcontrol_{it}$ are the control variable in each region and surrounding areas, respectively. $\beta_{i}$ represents the regression coefficient to be estimated, and $\mu_{i}$ is the personal effect, while the random disturbance term is defined $\varepsilon_{\mathrm{it}}$.

(5) $$\begin{array}{ll} {\mathrm{AGTFP}}_{\mathrm{it}} & =\beta_{0}+\beta_{1}w{\mathrm{AGTFP}}_{\mathrm{it}}+\beta_{2}{\mathrm{AST}}_{\mathrm{it}}+\beta_{3}{\mathrm{AST}}_{\mathrm{it}}^{2}+\beta_{4}{\mathrm{TL}}_{\mathrm{it}}+\beta_{5}{\mathrm{EI}}_{\mathrm{it}}+\beta_{6}{\mathrm{AST}}_{\mathrm{it}}\times {\mathrm{TL}}_{\mathrm{it}}+\beta_{7}{\mathrm{AST}}_{\mathrm{it}}\times {\mathrm{EI}}_{\mathrm{it}}+\beta_{8}{\mathrm{AST}}_{\mathrm{it}}^{2}\times {\mathrm{TL}}_{\mathrm{it}}\\ & +\beta_{9}{\mathrm{AST}}_{\mathrm{it}}^{2}\times {\mathrm{EI}}_{\mathrm{it}}+\beta_{10}\omega {\mathrm{AST}}_{\mathrm{it}}+\beta_{11}\omega {\mathrm{AST}}_{\mathrm{it}}^{2}+\beta_{12}{\omega \mathrm{TL}}_{\mathrm{it}}+\beta_{13}{\omega \mathrm{EI}}_{\mathrm{it}}+\beta_{14}\omega {(\mathrm{AST}}_{\mathrm{it}}\times {\mathrm{TL}}_{\mathrm{it}})+\beta_{15}\omega {(\mathrm{AST}}_{\mathrm{it}}\times {\mathrm{EI}}_{\mathrm{it}})\\ & +\beta_{16}\omega ({\mathrm{AST}}_{\mathrm{itit}}^{2}\times {\mathrm{TL}}_{\mathrm{it}})+\beta_{17}\omega ({\mathrm{AST}}_{\mathrm{itit}}^{2}\times {\mathrm{EI}}_{\mathrm{it}})+{\mathrm{control}}_{\mathrm{it}}+\omega {\mathrm{control}}_{\mathrm{it}}+\mu_{i}+\varepsilon_{\mathrm{it}} \end{array}$$

#### 3.3.3. The Explanatory Variables of the Spatial Durbin Model

Referring to Fang et al. [38], we set control variables in the spatial economic model as the following: (a) The level of economic development (pgdp) is expressed by the per capita GDP of each region. (b) Natural disaster (dis) is defined by the proportion of affected area to a sown area of crops. (c) The degree of agricultural mechanization (m) is expressed as the ratio of the total power of machinery to those employed in each agrarian sector. (d) Industrialization (ind) is defined by the value added by industry as a share of GDP. (e) Urbanization (urban) is represented by the proportion of the nonagricultural population in the total population. (f) Financial support for agriculture (FSA) is characterized by the ratio of agricultural financial expenditure to the sown area of crops. The statistical description of all variables above is described in Table 3.

### 3.4. Specification of Threshold Regression Model

To investigate the threshold effect of agricultural structure on the relationship between technological progress and AGTFP, this paper draws on the panel threshold effect test and threshold regression proposed by Hansen [41]. The study builds a threshold regression model in Equations (6) and (7) with the rationalization of agricultural structure (TL) as the threshold. In the equations, the subscript i represents each province, and subscript t represents the year. $\alpha_{i}$,$\chi_{\mathrm{it}}$ are the regression coefficient to be estimated. $TL_{\mathrm{it}}$ is the threshold variable; I(.) is the indicative function. $\delta_{i}$ and $\gamma_{i}$ are the threshold value, where the error term are $\varepsilon_{\mathrm{it}}$ and $\zeta_{\mathrm{it}}$.$\varepsilon_{\mathrm{it}}^{},\zeta_{\mathrm{it}}\sim \mathrm{iid}\;(0,\sigma^{2})$. The control variables in the threshold model are the same as the above spatial Durbin model. Section 3.3.3 for details is viewed.

(6) $$\begin{array}{ll} {\mathrm{AGTFP}}_{\mathrm{it}}^{} & =\alpha_{0}^{}+\alpha_{11}{\mathrm{AST}}_{\mathrm{it}}^{}\times {I(\mathrm{TL}}_{\mathrm{it}}^{}\leq \gamma_{1}^{})+\alpha_{12}{\mathrm{AST}}_{\mathrm{it}}^{}\times I(\gamma_{1}<{\mathrm{TL}}_{\mathrm{it}}^{}<\gamma_{2})+\alpha_{13}{\mathrm{AST}}_{\mathrm{it}}^{}\times I(\gamma_{2}\leq {\mathrm{TL}}_{\mathrm{it}}^{}<\gamma_{3})+\alpha_{14}{\mathrm{AST}}_{\mathrm{it}}^{}\times I(\gamma_{3}\leq {\mathrm{TL}}_{\mathrm{it}}^{})\\ & +\alpha_{2}\mathrm{control}+\varepsilon_{\mathrm{it}} \end{array}$$

(7) $$\begin{array}{ll} {\mathrm{ATFP}}_{\mathrm{it}}^{} & =\chi_{0}^{}+\chi_{11}{\mathrm{AST}}_{\mathrm{it}}^{}\times {I(\mathrm{TL}}_{\mathrm{it}}^{}\leq \delta_{1}^{})+\chi_{12}{\mathrm{AST}}_{\mathrm{it}}^{}\times I(\delta_{1}^{}<{\mathrm{TL}}_{\mathrm{it}}^{}<\delta_{2}^{})+\chi_{13}{\mathrm{AST}}_{\mathrm{it}}^{}\times I(\delta_{2}^{}\leq {\mathrm{TL}}_{\mathrm{it}}^{}<\delta_{3}^{})+\chi_{14}{\mathrm{AST}}_{\mathrm{it}}^{}\times I(\delta_{3}^{}\leq {\mathrm{TL}}_{\mathrm{it}}^{})\\ & +\chi_{2}\mathrm{control}+\zeta_{\mathrm{it}} \end{array}$$

## 4. Results and Discussion

### 4.1. AGTFP in China

**Figure 1:**
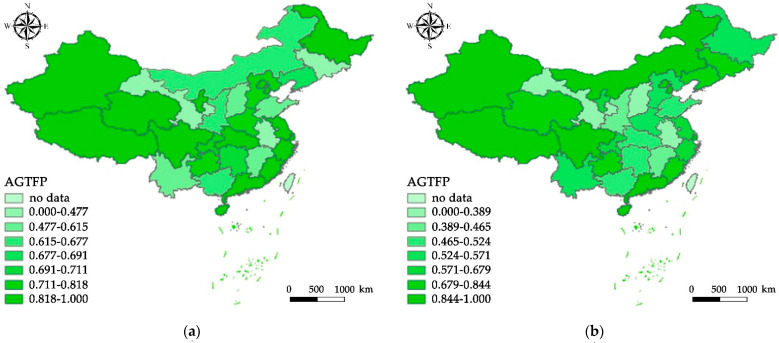

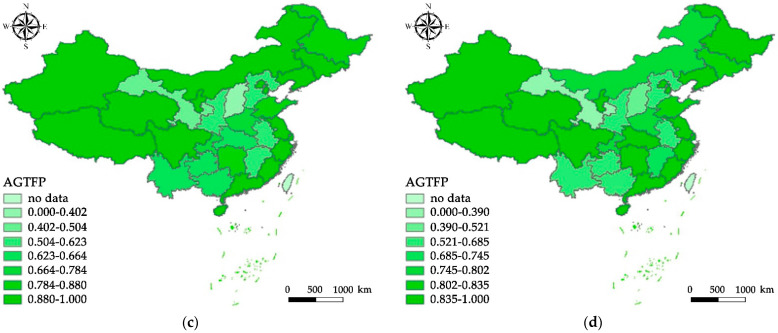
Distribution of the agricultural GTFP in China. (**a**) Distribution of the Agricultural GTFP in 2005; (**b**) Distribution of the Agricultural GTFP in 2010; (**c**) Distribution of the Agricultural GTFP in 2015; (**d**) Distribution of the Agricultural GTFP in 2019.

AGTFP from China from 1998 to 2019 is calculated using MaxDEA 8.0 software by Ruiwo Meidi Software Ltd., Beijing China. As shown in Figure 1, Figure 1a depicts the agricultural GTFP in 2005, the last year of China’s Tenth Five-Year Plan (2001–2005). Similarly, Figure 1b illustrates the distribution of the AGTFP during China’s Eleventh Five-Year Plan (2006–2010), Figure 1c depicts the distribution of the agricultural GTFP during China’s Twelfth Five-Year Plan (2011–2015). Figure 1d shows the distribution of the AGTFP during China’s Thirteenth Five-Year Plan (2016–2020).

Crediting the continuous strengthening of agricultural pollution control and ecological environment protection in China, the AGTFP in Eastern, Central, and Western China all experienced fluctuating improvements, from 0.61845, 0.3213, and 0.5195 in 1998 to 0.9339, 0.649035, and 0.8376 in 2019 respectively. (The Eastern region includes Beijing, Tianjin, Hebei, Liaoning, Shanghai, Jiangsu, Zhejiang, Fujian, Shandong, Guangdong, and Hainan; the Central region includes Jilin, Shanxi, Heilongjiang, Anhui, Jiangxi, Henan, Hubei, Hunan, Inner Mongolia, and Guangxi; the Western region Including Xinjiang, Gansu, Shaanxi, Ningxia, Sichuan, Chongqing, Guizhou, Yunnan, Tibet, Qinghai). This result is supported by Liu et al. and Wang et al. [7,42]. During the Tenth Five-Year Plan period, the government began to implement a preferential agricultural policy of reducing or exempting agricultural taxes, which led to the growth of agricultural GTFP. Later, the government increased the total direct subsidies for farming materials and increased support for agriculture during the Eleventh Five-Year Plan period. The growth rate of AGTFP is relatively stable. Recently, the government promoted the development of industrialization and urbanization and guided agriculture to embark on a modern agricultural path featuring “efficient output, product-safety, resource-conservation, and environmental friendliness” during the Twelfth Five-Year Plan period.

Furthermore, AGTFP in China has solid spatial agglomeration and spatial heterogeneity. AGTFP in the Eastern region is highest, followed by the Western part, and is the lowest in the Central region. This conclusion is supported by authoritative scholars such as Gucheng [43]. The Eastern region, economically more developed areas, has achieved a higher efficiency of green agricultural production, which has a strong awareness of greener production, infrastructure investment, and rich agrarian talent resources, as well as an extension of agricultural science and technology. The shift of heavily polluting industries from the Eastern region to the Central part, the major grain-producing area, has caused prolonged conflict between the shrinking of the resource capacity of the Central region and the local development. The Western region has significant environmental power, with a sparse population and pollutant emissions [44].

### 4.2. Results and Discussion of the Spatial Durbin Model

Preventing the emergence of endogenous or missing variables, we choose GTFP_t−1_ and w*GTFP_t−1_ to represent the temporal and spatial lag terms, respectively, and adopt a more commonly spatial SDM panel estimation method. The *p*-value of the Hausman test of the spatial Durbin model is 0.000, which indicates that the fixed-effect models should be chosen. In addition, after analyzing the R2 and LogL values, it is believed that constructing the spatial and time double fixed-effects SDM model is appropriate. We report only the regression results after selection, as shown in Table 4. Column (a) is the result of the spatial Durbin model without considering the agricultural structure, while Column (b) believes the farm structure, for which we can drive the following conclusion:

Firstly, the spatial autoregressive coefficients of AGTFP are 0.715, 0.792, and a significance all at the 1% level, indicating that the agricultural GTFP exerts a positive spatial spillover effect, which will be approved by Yu et al. [45]. In other words, the agricultural GTFP of a province, closely related to neighboring regions in geography, is an open system, perfectly demonstrating “Frankness near Melbourne black”. More attention should be paid to spatial arrangement. Upgrading the agricultural structure significantly enhances such a spatial autocorrelation.

Secondly, local AST is positively correlated with AGTFP but negatively when the provinces are adjacent when AST^2^ is negatively correlated with local AGTFP but positively in a neighboring region, which indicates that technological progress and AGTFP are in an obviously inverted U-shaped relationship locally but in U-shaped ties in the surrounding area. The AGTFP system is a nonlinearity regime. Technological progress starts from existing or potential problems in the local farming system. Therefore, any technological advancement will undoubtedly give priority to supporting the local agricultural system. In the early stage, technological progress always effectively solves the obstacles of the agricultural efficiency system and increases the marginal contribution to the local AGTFP. However, hindered by backward supporting facilities and weak technical reproducibility, the introduction of technological progress has aggravated the pressure on the farm system of the surrounding provinces, squeezing other truly suitable resource inputs, impeding the improvement of AGTFP instead. In addition, technological progress is a dynamic process. New technologies will “sink” all the original equipment that matches the old technologies [46]. When technological advancement cannot solve the issues of the agricultural green efficiency system, the time-lapse path, technological dependence, and related costs will simultaneously drag down the process of green agricultural efficiency. Technological progress promotes the local AGTFP at first and then hinders it. Meanwhile, the introduced technological progress has a specific adaptation process and spatial friction to improve AGTFP.

Thirdly, compared to Column (a), it is evident that the coefficient of AST and AST^2^ is higher in Column (b), which indicates that the upgrading of agricultural structure enhances not only the inverted U-shaped relationship between the technological progress and AGTFP at local but the U-shaped relationship at surrounding. The AGTFP system is far from equilibrium. The U-shaped relationship between technological progress and AGTFP has strengthened with the agrarian structure that may lie in these aspects. On the one hand, the subdivision and adjustment of the proportion of the farm sector mean the flow and re-allocation of elements. The original intention of the adjustment of the agricultural sector is to improve the agricultural GTFP and achieve modern agriculture. High value-added industries and products occupy favorable positions, leading subsectors to realize the refinement of agricultural production and the specialization of production equipment, catalyze the spillover and diffusion of agricultural technical efficiency, increase production while reducing carbon emissions, and drive the green all-factors of the entire industry productivity.

On the other hand, such an adjustment in agricultural structure also has cost and spatial friction coefficients. The marginal energy of the upgrading of the agrarian system is diminishing. When the marginal energy is less than the marginal cost, it will inevitably hinder the growth of the contribution of technological progress to AGTFP. In addition, the spatial friction of changes in the farming structure has delayed technological progress in satisfying AGTFPs in neighboring provinces.

### 4.3. Results and Discussion of the Threshold Regression Model

It detects the threshold regime dependent variable by bootstrap sampling 300 times. Only when a threshold value is the rationalization of agricultural structure but not its advancement could technological progress pass the single threshold test under the significant level of 10% for AGTFP or agricultural TFP, which indicates that technological progress has a single threshold effect. It is proven again that the AGTFP system is subject to the dissipative structure theory, with the characteristics such as open, nonlinear, and far from equilibrium. It can be seen in Table 5 that the threshold values of agricultural TFP (ATFP) and AGTFP are −5.179.

The estimation results of Equations (6) and (7) are shown in Table 6 as Column (c) and Column (d). When the rationalization of agricultural structure value is lower than the single threshold, every 1 unit increase in technological progress leads to an increase of 1.608 in agricultural TFP or an increase of 0.314 in AGTFP. While the rationalization of agrarian structure value crosses the threshold, every 1 unit increase in technological progress leads to an increase of 1.742 in agricultural TFP and an increase of 0.38 in AGTFP. The growth rate of agricultural TFP is lower than that of AGTFP. Therefore, some insights can be deducted from these results, given that the estimates are robust enough. It seems that the contribution of technological progress to AGTFP or TFP would be enhanced as the threshold of industrial structure rationality increases. The agrarian technology also promotes agricultural TFP to a greater extent than AGTFP.

In contrast, the rationalization of agricultural structure crosses the single threshold, and technological progress is more conducive to promoting the growth of the farm AGTFP. This finding reveals that the AGTFP and TFP align with the coordination degree among agricultural subsectors. In contrast, the change and refinement of the farm production structure will trigger a technology-induced effect, increase the added value of products, and reduce carbon emissions. The realization of the division of departments and products and the improvement of production efficiency have led to a new round of replacement between industry and technology, and the requirements for energy conservation and emission reduction have become more apparent, which makes the agrarian structure affect the growth of AGTFP by changing technical efficiency.

## 5. Conclusions

Existing literature lacks an in-depth consideration of the particularity of the agrarian system when analyzing the effect of agricultural structure on the relationship between technological progress and agricultural green total productivity in China. Based on the dissipative structure theory, we first measure the technological progress by the Solow growth model, calculate the AGTFP by nonradial and nonoriented SBM, respectively, then divide the upgrading of agricultural structure into two aspects: rationalization and advanced. Next, we develop a spatial Durbin model to identify the nonlinear impact of technological progress spillover on AGTFP with the agricultural structure as an influencing factor and then construct a threshold panel model to explore the threshold effect of technological progress on AGTFP when an agricultural structure is used as a threshold variable and further discuss the difference between agricultural GTFP and TFP. The following conclusions have been made: (1) From 1998 to 2019, the AGTFP in China experienced fluctuating improvements. Furthermore, the AGTFP in China has solid spatial agglomeration and spatial heterogeneity. AGTFP in the Eastern region is highest, followed by the Western part, and is the lowest in the Central region. (2) The progress of the independent technologies initially promotes the growth of AGTFP and then makes AGTFP decline. By contrast, the progress of the technologies in introduction initially weakened the growth of AGTFP and then promoted AGTFP. Further, upgrading agricultural structure enhances not only the inverted U-shaped relationship between technological progress and AGTFP locally but also the U-shaped relationship in the surrounding area. (3) Technological progress has a single threshold effect on AGTFP with the rationalization of agricultural structure as the threshold value. The contribution of technological progress to agricultural GTFP or TFP would be enhanced as the threshold of industrial structure rationality increases, but the agricultural technology promotes agricultural TFP to a greater extent than AGTFP. In a word, the effect of agricultural structure on the relationship between technological progress and agricultural green total productivity in China is subject to the dissipative structure theory with a series of characteristics, i.e., an opening system, being far from equilibrium, a nonlinearity regime.

The following policy implications can be obtained from the theoretical and empirical discussion: Firstly, China should do its best to strengthen independent agricultural technologies and innovation, change the mode of economic development and strengthen the guidance of technological progress to boost the growth of AGTFP. The formulation of technology policies should incorporate the staged characteristics in China, grasp the macroscopic environment of technologies, recognize its evolutionary course, and use the law of technologies to maximize the quality of AGTFP. Meanwhile, the access threshold of agricultural technology should be further lowered, and an appropriate technology screening system should be established rigorously. For example, the negative list management of inappropriate agricultural technology, listing all actual demand and development direction of agricultural technology in the region, should be promoted. Regional decision-makers must conduct in-depth research on regional agriculture and formulate correct agricultural technology and green agricultural regulations based on the bottlenecks in the agricultural transformation. Secondly, focus on upgrading the green agrarian structure and developing a resource-recycling green agriculture model. The government drives farmers to create industrialized operations, extends the industrial chain of green agricultural products, and promotes the improvement of the overall quality and market competitiveness of relevant green agricultural product manufacturers. At the same time, a practical integrated development system for the primary, secondary, and tertiary industries in rural areas should be built vigorously to reduce the structural proportion of high-energy-consuming industries, implement intensive management of agricultural resources, attach importance to local ecological protection, adhere to clean production, then realizes the maximization of resource utilization.

For this moment, our analysis is silent about proving the contribution of the different biased agricultural technologies to AGTFP or the horizontal comparison with other developing countries. These are some lacking that we will continue to follow up for future research. Nevertheless, we believe that the discussion in this paper has an excellent value for the economies undergoing a similar development stage to China to improve agricultural technology and transform agricultural structural models while catching up with AGTFP, achieving sustainable agrarian methods.

## Figures and Tables

**Table 1:** Indicator index system for agricultural GTFP in China.

Indicator	Index	Definition	Unit
Input indicators	Labor	Number of employees in agriculture	10,000 individuals
	Land	The planting area of crops	1000 HA
	Chemical fertilizer	Amount of agricultural chemical fertilizer application	10,000 tons
	Mechanical power	Total power of agricultural machinery	10,000 kW
	Electric power	Rural electricity consumption	10,000 kWh
Output indicators	Desired output	The gross production of agriculture	100 million yuan
	Undesired output	Agricultural carbon emissions	10,000 tons

**Table 2:** The Global Moran’s index of Core variables from 1998 to 2019 in China.

Variable	TL		EI		AST		AGTFP	
Year	Moran’s I	*p*-Value	Moran’s I	*p*-Value	Moran’s I	*p*-Value	Moran’s I	*p*-Value
1998	0.276 ***	0.004	0.208 **	0.018	0.143 **	0.043	0.088 ***	0.000
1999	0.287 ***	0.003	0.216 **	0.015	0.151 **	0.037	0.132 ***	0.000
2000	0.280 ***	0.003	0.227 **	0.012	0.154 **	0.035	0.104 ***	0.000
2001	0.279 ***	0.003	0.240 ***	0.009	0.159 **	0.035	−0.003 ***	0.000
2002	0.276 ***	0.004	0.247 ***	0.008	0.163 **	0.034	−0.003 ***	0.000
2003	0.284 ***	0.003	0.250 ***	0.007	0.202 **	0.016	−0.003 ***	0.000
2004	0.291 ***	0.002	0.291 ***	0.003	0.173 **	0.030	−0.024 ***	0.000
2005	0.286 ***	0.003	0.317 ***	0.001	0.175 **	0.029	0.004 ***	0.000
2006	0.286 ***	0.003	0.314 ***	0.001	0.173 **	0.032	0.012 ***	0.000
2007	0.274 ***	0.004	0.312 ***	0.001	0.153 **	0.046	0.110 ***	0.000
2008	0.273 ***	0.004	0.317 ***	0.001	0.129 *	0.071	0.077 ***	0.000
2009	0.261 ***	0.005	0.311 ***	0.001	0.141 *	0.057	0.095 ***	0.000
2010	0.241 ***	0.008	0.314 ***	0.001	0.167 **	0.035	0.050 ***	0.000
2011	0.298 ***	0.002	0.364 ***	0.000	0.166 **	0.036	0.050 ***	0.000
2012	0.295 ***	0.002	0.357 ***	0.000	0.168 **	0.036	0.072 ***	0.000
2013	0.255 ***	0.006	0.330 ***	0.001	0.159 **	0.042	0.140 ***	0.000
2014	0.253 ***	0.006	0.322 ***	0.001	0.148 *	0.051	0.097 ***	0.000
2015	0.253 ***	0.006	0.329 ***	0.001	0.155 **	0.046	0.053 ***	0.000
2016	0.247 ***	0.007	0.342 ***	0.000	0.157 **	0.045	0.083 ***	0.000
2017	0.261 ***	0.005	0.354 ***	0.000	0.159 **	0.042	0.032 ***	0.006
2018	0.250 ***	0.006	0.338 ***	0.001	0.160 **	0.042	−0.015 ***	0.000
2019	0.255 ***	0.005	0.254 ***	0.006	0.153 **	0.047	−0.151 ***	0.000

Note: The variables marked with *, **, and *** are significant at the significance levels of 10%, 5%, and 1%, respectively.

**Table 3:** The statistical description of the variables.

Variables	Observations	Mean	Std. Dev.	Min	Max
AGTFP	682	0.766	0.246	0.217	1
ATFP	682	0.917	0.141	0.447	1
AST	682	1.272	0.177	0.986	2.006
TL	682	−6.236	1.089	−8.180	−3.464
EI	682	−0.003	0.001	−0.004	0.002
pgdp	682	31,873.85	27,102.14	2342	164,220
dis	682	0.239	0.163	0	0.936
ind	682	0.373	0.105	0.068	1.284
urban	682	46.511	17.815	13.8	89.6
m	682	3.170	2.482	0.354	35.366
FSA	682	1.022	0.091	0.417	2.077

**Table 4:** The estimation results of the spatial Durbin model.

Variables	(a)	(b)	Variables	(a)	(b)
AGTFP_t−1_	0.871 *** (5.84)	0.764 *** (31.61)	W*AGTFP_t−1_	0.715 *** (5.20)	0.792 *** (22.28)
AST	0.422 (0.43)	1.258 *** (3.51)	W*AST	−0.171 (−1.23)	−1.764 *** (−3.02)
AST^2^	−0.393 *** (−3.31)	−0.679 *** (−2.96)	W*AST^2^	0.111 * (1.68)	1.331 *** (4.96)
TL		−0.583 *** (−5.58)	W*TL		−0.016 (−0.15)
EI		559.95 *** (4.88)	W*EI		−936.115 (−4.21)
AST*TL		−0.146 (−0.75)	W*(AST*TL)		0.101 (1.08)
AST*EI		21.057 *** (4.76)	W*(AST*EI)		638.642 *** (3.76)
AST^2^*TL		0.05 ** (1.39)	W*(AST^2^*TL)		−0.399 ** (−2.207)
AST^2^*EI		−1.94 * (−1.22)	W*(AST^2^*EI)		−21.346 (−0.42)
Ln(pgdp)	−0.004 (−0.8)	−0.007 (−0.31)	W*ln(pgdp)	−0.001 (−1.45)	0.019 (−0.66)
dis	0.019 (0.66)	0.012 (0.3)	W*dis	0.003 (0.07)	−0.062 (−0.85)
FSA	0.21 *** (4.98)	0.1 * (1.82)	W*FSA	−0.06 (−0.8)	−0.138 (−1.36)
urban	0.000 (0.38)	−0.003 * (−1.95)	W*urban	0.002 ** (1.97)	0.004 ** (2.38)
ind	−0.043 (−0.79)	0.084 (0.99)	W*ind	0.156 (1.59)	0.84 *** (6.14)
m	−0.003 (−1.33)	0.007 ** (2.23)	W*m	0.001 (0.25)	−0.004 (−0.79)
cons	−0.978 *** (−3.82)	2.31 ** (2.31)			
R-sq	0.621	0.859	Log-likelihood	474.804	675.44

Note: *, **, and *** are respectively significant at the levels of 10%, 5%, and 1%. The *t* value is in parentheses.

**Table 5:** The test of a threshold effect.

Dependent Variable	Independent Variable	Threshold Variable	Model	Threshold	F-Stat	Prob	BS	Critical Value		
								10%	5%	1%
ATFP	AST	TL	Single	−5.179	152.49	0.000	300	52.918	65.419	89.525
			Double	−5.215 −6.691	32.67	0.49	300	74.335	87.735	123.104
			Triple	−7.292	30.83	0.597	300	63.207	71.979	90.221
AGTFP	AST	TL	Single	−5.179	151.94	0.000	300	64.831	77.391	107.313
			Double	−6.2015 −7.273	45.91	0.49	300	74.335	87.735	123.104
			Triple	−7.83	30.83	0.597	300	63.207	71.979	90.221

**Table 6:** The estimation results of the threshold regression model.

Variables	(c)	(d)	Variables	(c)	(d)
AST(Th-1)	1.608 *** (3.60)	0.314 * (1.71)	dis	−0.002 (−0.05)	−0.022 (−0.84)
AST(Th-21)	1.742 *** (4.06)	0.38 * (1.9)	ind	0.329 ** (2.07)	0.184 ** (2.12)
FSA	0.061 *** (2.7)	0.014 *** (2.59)	m	0.035 *** (3.63)	0.002 (1.47)
R-sq	0.507	0.381	pgdp	0.004 ** (3.05)	0.001 (1.40)
Number of obs	682	682			

Note: *, **, and *** are respectively significant at the levels of 10%, 5%, and 1%. The *t* value is in parentheses.

## Data Availability

Not applicable.
